# An Efficient Chronic Unpredictable Stress Protocol to Induce Stress-Related Responses in C57BL/6 Mice

**DOI:** 10.3389/fpsyt.2015.00006

**Published:** 2015-02-02

**Authors:** Susana Monteiro, Susana Roque, Daniela de Sá-Calçada, Nuno Sousa, Margarida Correia-Neves, João José Cerqueira

**Affiliations:** ^1^Life and Health Sciences Research Institute (ICVS), School of Health Sciences, University of Minho, Braga, Portugal; ^2^ICVS/3B’s Research Group – PT Government Associate Laboratory, Braga, Portugal

**Keywords:** chronic stress, CUS, neuropsychiatric disorders, immune dysfunction, anxiety, depressive-like behavior, social defeat

## Abstract

Exposure to chronic stress can have broad effects on health ranging from increased predisposition for neuropsychiatric disorders to deregulation of immune responses. The chronic unpredictable stress (CUS) protocol has been widely used to study the impact of stress exposure in several animal models and consists in the random, intermittent, and unpredictable exposure to a variety of stressors during several weeks. CUS has consistently been shown to induce behavioral and immunological alterations typical of the chronic stress-response. Unfortunately C57BL/6 mice, one of the most widely used mouse strains, due to the great variety of genetically modified lines, seem to be resistant to the commonly used 4-week-long CUS protocol. The definition of an alternative CUS protocol allowing the use of C57BL/6 mice in chronic stress experiments is a need. Here, we show that by extending the CUS protocol to 8 weeks is possible to induce a chronic stress-response in C57BL/6 mice, as revealed by abrogated body weight gain, increased adrenals weight, and an overactive hypothalamic–pituitary–adrenal axis with increased levels of serum corticosterone. Moreover, we also observed stress-associated behavioral alterations, including the potentiation of anxious-like and depressive-like behaviors and a reduction of exploratory behavior, as well as subtle stress-related changes in the cell population of the thymus and of the spleen. The present protocol for C57BL/6 mice consistently triggers the spectrum of CUS-induced changes observed in rats and, thus, will be highly useful to researchers that need to use this particular mouse strain as an animal model of neuropsychiatric disorders and/or immune deregulation related to CUS.

## Introduction

Stressful life events can be triggering factors of numerous neuropsychiatric disorders namely anxiety, depression, and dementia (1), and many of these are accompanied by immune dysfunction (2). Moreover, prolonged-stress-induced immune dysfunction itself is regarded as a contributing factor for the effects of stress on health (3). In contrast with chronic stress, the acute stress-response is a beneficial event since it is an alarm reaction that prepares the body to a possible threat. This response is characterized by the secretion of stress mediators, such as glucocorticoids and epinephrine, which allows the stability of body function by adaptation to the stressor (4). However, when this response persists in time, it might render the system unable to cope with the stressor, ultimately leading to chronic-stress-associated illness.

Neuropsychiatric alterations are the most widely described effects of chronic stress exposure and include anxious-like behavior (5–8), depressive-like behavior (9, 10), and cognitive deficits (5, 11–14). However, the effects of chronic stress are not only limited to behavioral changes. Immune cells express receptors for glucocorticoids and catecholamines (15, 16), which can lead to alterations in gene transcription in response to stress (17). In fact, it is generally accepted that chronic-stress-associated changes in the immune system alter the vulnerability to infectious disease and auto-immunity (18).

Stress exposure variables, such as duration, intensity, and predictability, explain the spectrum of differential responses to stress but ultimately, the threshold in which the stress-response switches from physiological to deleterious is also dependent on neuroendocrine, neurochemical, and genetic factors that are responsible for individual differences in stress perception and response (19). Having this in mind, it seems logical that for the use of animal models, the chronic stress protocol needs to be adjusted to the animal species and even the strain used.

The most commonly used unpredictable chronic stress paradigms are the unpredictable chronic mild stress (uCMS) and the chronic unpredictable stress (CUS). Although both terms, uCMS and CUS, tend to be used indiscriminately nowadays and that both protocols are widely used to study depression, the original purpose for which they were generated was quite distinct. uCMS paradigm have been long used to model depression, and consists in the continuous exposure of animals to stressful situations, usually for at least 4 weeks, including some stressors that involve water and/or food deprivation. In contrast, CUS was originally used to study mechanisms underlying the stress-response and involves the intermittent exposure to a daily stressful stimulus, lasting at least 4 weeks, being one of the main advantages of this protocol the absence of stressors that interfere with water and/or food deprivation, which might better mimic everyday life stress.

Although rats are widely used as animal models of depression and other stress-related disorders, mice present advantages such as the availability of numerous genetically modified strains like transgenic and KO mice and the lower maintenance costs when compared to rats. Unfortunately, the most widely used inbreed strain of genetically modified mice, the C57BL/6, seems to be less vulnerable to stress than other mouse strains (20–25).

Our aim was to develop an improved CUS protocol to be used in C57BL/6 mice. In order to do so we modified the standard CUS protocol by including social defeat stress as one of the stressors and extending its duration to 8 weeks. By comparing the neuroendocrine, behavioral, and immune changes induced by the unmodified 4-week long CUS exposure and the optimized 8-week long CUS protocol we, herein, show the advantages of later for C57BL/6 mice.

## Materials and Methods

### Animals

Male C57BL/6 mice (C57BL/6J JAX™ mice strain) were purchased from Charles River (Charles River Laboratories, Barcelona, Spain) and housed (five animals per cage) under standard laboratory conditions (12 h light/12 h night cycles (8 h/20 h), 22–24°C, relative humidity of 55% and *ad libitum* access to water and food. All procedures were carried out in accordance to EU directive 2010/63/EU and Portuguese national authority for animal experimentation, Direção Geral de Veterinária (ID:DGV9457) guidelines on animal care and experimentation.

### Chronic unpredictable stress paradigm

One group of C57BL/6 animals was exposed to 4 weeks of CUS and compared to a control group that was subjected to gentle handling, twice a week, for the same period. Another group was exposed to 8 weeks of CUS and compared to other control group that was subjected to gentle handling, twice a week, for the same period. Mice were 8-week old when the CUS protocol was initiated. Each group consisted of 10–15 male C57BL/6 mice. We run two independent experiments to confirm our findings: data from the first, representative of our findings, are presented in the main paper, whereas data from the second experiment are shown as supplementary data (Figure S1 and Table S1 in Supplementary Material).

Briefly, the CUS paradigm consisted in exposure, once daily, to one of the following aversive stressors: **restraint** – mice were placed in a 50 ml plastic tube (Falcon) with openings in both sides for breathing, for 1 h; **shaking** – groups of five mice were placed in a plastic box container and placed in an orbital shaker for 1 h at 150 rpm; **social defeat** – mice were introduced in a cage of an aggressive mice and after being defeated, they were placed in a transparent and perforated plastic container, to avoid further physical contact, inside the resident homecage for 30 min (26); **hot air stream** – mice were exposed to a hot air stream from a hairdryer for 10 min; **overnight illumination** – mice were exposed to regular room light during the night period; **inverted light cycle** – regular room light was off during daytime and on during nighttime for 2 days; **tilted cage** – homecages were tilted in a 45° angle during 1 h. Stressors were presented in a random order in an unpredictable fashion (see Table 1). The stressors distribution for the group submitted to 4 weeks of CUS is a truncated version of Table 1. Body weight was monitored once a week and *post-mortem* thymus and adrenal weight were recorded.

**Table 1:** **Example of stressors distribution**.

	Mon	Tue	Wed	Thu	Fri	Sat	Sun
Week 1	BW hot drier	Shaking	Restraint	Social defeat	Restraint	Restraint	Tilted cage
Week 2	BW restraint	Shaking	Social defeat	Restraint	Shaking	Social defeat	Restraint
Week 3	BW restraint	Social defeat	Restraint	BC-*zenith*	Social defeat	Restraint	Shaking
Week 4	BW	EPM	FST	Shaking	Restraint	Shaking	Hot drier
	BC – *nadir*	OF	TST	
	Social defeat	Restraint	Social defeat	
Week 5	BW restraint	Sacrifice 4 weeks tilted cage	Cytometry hot drier	Restraint	Social defeat	Inverted light	Inverted light
Week 6	BW hot drier	Shaking	Restraint	Inverted light	Overnight illumination	Restraint	Hot drier
Week 7	BW restraint	Social defeat	Restraint	BC-*zenith*	Shaking	Restraint	Overnight illumination
Week 8	BWBC-*nadir*	EPM OF Restraint	FST TST Social defeat	MWM shaking	MWM restraint	MWM restraint	MWM shaking
Week 9	BW	Sacrifice 8 weeks	Cytometry				

*BW, body weight measurement; BC, blood collection; EPM, elevated-plus maze; OF, open field; FST, forced swimming test; TST, tail-suspension test; MWM, Morris water maze test*.

### Corticosterone quantification

Blood was collected through the tail by venopuncture within a maximum 120 s period since removal of each mouse from its homecage to the end of blood collection. Sera were separated by centrifugation at 13000 rpm, during 5 min and stored at −80°C. Serum corticosterone levels were measured on sera collected at *nadir* phase (9:00 a.m.) and at *zenith* phase (8:00 p.m.) using a commercial radioactive immunoassay kit (MP Biomedicals, CA, USA).

### Behavioral assessment

Mice were transported and left for habituation to the testing room for 1 h prior to the behavioral test. The order of the behavioral tests was: elevated-plus maze (EPM) and open field (OF) (Day 1), forced swimming test (FST) and tail-suspension test (TST) (Day 2), and Morris water maze (MWM) (Day 3–7).

#### Elevated-plus maze

Anxious-like behavior was assessed using the EPM test (27). Briefly, this test consists on placing each mouse in the hub of a plus-like apparatus elevated 72.4 cm from the floor, with two opposing open arms (50.8 cm × 10.2 cm) and two opposing closed arms (50.8 cm × 10.2 cm × 40.6 cm) (ENV560; Med Associates, Inc., Vermont, USA) and letting the animal freely explore it for 5 min. Time in the open arms and in the closed arms was used as a behavioral parameter of anxious-like behavior. EPM data from one animal from each group were not included in the analysis due to failure of the video recording system.

#### Open field

Locomotor and exploratory activities were assessed using the OF. Each mouse was left in the center of a squared arena (43.2 cm × 43.2 cm), which the mouse was free to explore for 5 min. This arena is equipped with infrared beams for activity detection (Med Associates, Inc., Vermont, USA). Data were collected using the activity monitor software (Med Associates, Inc., Vermont, USA). Distance traveled was used as a measure of locomotor activity and the number of vertical counts as a measure of exploratory activity.

#### Forced swimming test and tail-suspension test

Depressive-like behavior was assessed through the FST as described by Ref. (28) and through the TST (29). Briefly, in the FST each mouse was placed in an inescapable transparent cylindrical tank filled with water (±24°C), for 6 min. In the TST, each animal was suspended by the tip of its tail for 6 min. The activity of each mouse, in both tests, was recorded using a video camera. Latency (time to the first stop), mobility and immobility times were scored manually by an investigator blind to the experimental conditions, using Etholog 2.2 software (30), and used as a measure of behavioral despair. TST data from one animal were not included in the analysis due to failure of the video recording system.

#### Morris water maze

In order to assess spatial reference memory, mice were tested in a white circular pool (170 cm diameter) filled with water (24–25°C) placed in a dimly lit room. Spatial cues were placed in the walls around the pool (square, stripes, triangle, and a cross). The pool was divided in four imaginary quadrants and a hidden transparent platform was placed in one of the quadrants. Data were collected by a fixed camera placed in the ceiling and connected to a video-tracking system (Viewpoint, Champagne-au-Mont-d’Or, France).

Mice had to learn the position of a hidden platform over a period of 4 days. In each day, mice were placed facing the wall of the pool at different quadrants (north, west, south, and east) as a starting point for each trial. Each trial was completed whenever the mouse reached the platform or when 120 s elapsed. Latency to reach the platform (escape latency) was recorded for each trial during the 4 days.

In the fifth day, the platform was removed and a single trial of 60 s was performed (probe trial). The percentage of time that each mouse swam in each quadrant was recorded to confirm the acquisition of platform location through reference memory.

### Flow cytometry

Thymus and spleen (8–10 animals per group) were dissected and homogenized in supplemented Dulbecco’s modified eagle medium (DMEM) with 10% heat inactivated FCS, 10 mM HEPES buffer, 1 mM sodium pyruvate, 2 mM l-glutamine, 50 μg/mL streptomycin, and 50 U/mL penicillin (all from Invitrogen, CA, USA) in order to obtain single-cell suspensions. Splenic erythrocytes were depleted by incubating for 5 min with a hemolytic solution (155 mM NH_4_Cl, 10 mM KHCO_3_, pH 7.2). To analyze the main cell populations in the thymus, the cells (1 × 10^6^ cell) were stained with APC anti-mouse CD3 (clone 145-2C11, Biolegend, San Diego, CA, USA), V450 anti-mouse CD4 (clone RM4-5, BD Pharmingen, San Jose, CA, USA), and V500 anti-mouse CD8 (clone 53-6.7, BD Pharmingen, San Jose, CA, USA). Splenocytes (1 × 10^6^ cell) were stained with APC anti-mouse CD3 (clone 145-2C11, Biolegend, San Diego, CA, USA) for T-lymphocytes, PE.Cy5.5 anti-mouse CD19 (clone 6D5, Biolegend, San Diego, CA, USA) for B-lymphocytes, V450 anti-mouse CD4 (clone RM4-5, BD Pharmingen, San Jose, CA, USA) for T helper cells, V500 anti-mouse CD8 (clone 53-6.7, BD Pharmingen, San Jose, CA, USA) for T cytotoxic cells, and FITC anti-mouse NK1.1 (clone PK136, Biolegend, San Diego, CA, USA) for natural killer cells. To analyze myeloid cell populations, splenocytes (1 × 10^6^ cell) were stained with PE anti-mouse CD11b (clone M1/70, Biolegend, San Diego, CA, USA), and PE.Cy7 anti-mouse Gr1 (clone RB6-8C5, Biolegend, San Diego, CA, USA). Cells were first gated for singlets (FSC-H vs. FSC-A) and viable cells (FSC-H vs. SSC-H). Myeloid cells were selected using the gating strategy described previously (31). Briefly, myeloid cells were gated as CD11b^+^ cells excluding the NK1.1^+^ cells. Macrophages/dendritic cells were selected as the population Gr1^+^SSC^low^, neutrophils were selected as Gr1^+^SSC^high^ and eosinophils as Gr1^−^SSC^high^ (Figure 7C). After staining cells were fixed in 2% paraformaldehyde for 20 min. Cell surface staining was acquired (100,000 events) in an eight-color LSRII flow cytometer (BD, Pharmingen, San Jose, CA, USA) and analyzed with FlowJo software version 7.6.4.

### Statistical analysis

All values were calculated as means ± SEM. Kolmogorov–Smirnov normality test was used to analyze if values departed from an approximate Gaussian distribution. Body weight, serum corticosterone levels, and reference memory task data were compared between groups using ANOVA repeated-measures on the average results of each week/phase/day, respectively. When the main effect was significant, *post hoc* Bonferroni test was performed in order to assess whether means differed significantly from each other. For all the other data, the differences among groups were analyzed using Student’s *t*-test. Differences were considered significant if *p* < 0.05. Statistical analysis was performed with Graphpad Prism version 5.0b (La Jolla, San Diego, USA).

## Results

### Biometric parameters and corticosterone measurements

**Figure 1:**
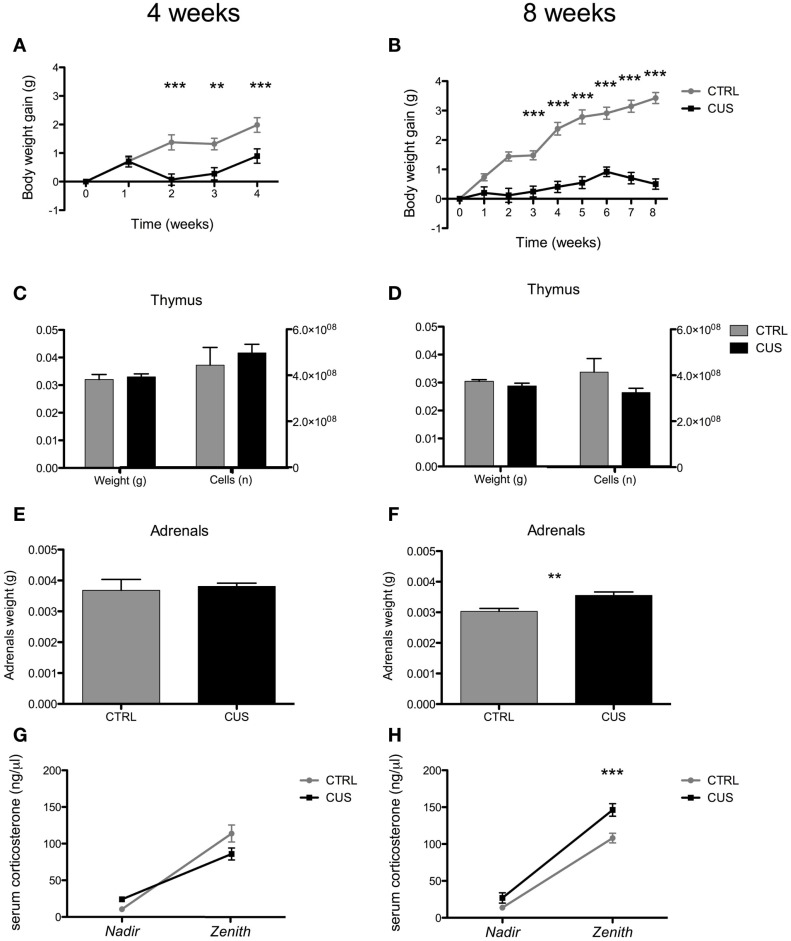
**Effect of 4 weeks vs. 8 weeks of CUS on biometric parameters**. Body weight gain for animals submitted to 4 **(A)** and 8 **(B)** weeks of CUS. Thymus weight and cellularity after exposure to 4 **(C)** and 8 **(D)** weeks of CUS. Adrenals weight after 4 **(E)** and 8 weeks of CUS **(F)**. Corticosterone levels in the serum of animals from the group submitted to 4 **(G)** and 8 **(H)** weeks of CUS. Each bar/point represents the mean ± SEM from 10 animals per group. **p < 0.01, ***p < 0.001.

Body weight gain, *post-mortem* thymus, and *post-mortem* adrenal weight and serum levels of corticosterone were monitored to control for stressors efficacy (Figure 1; Figure S1 in Supplementary Material). In the group submitted to the 4-week protocol of CUS, both time [*F*_(4,72)_ = 23.85; *p* < 0.0001] and exposure to CUS [*F*_(1,18)_ = 11.94; *p* = 0.003] had a significant impact on body weight (Figure 1A). Moreover, there was a significant interaction between these factors [*F*_(4,72)_ = 23.85; *p* < 0.0001] with stressed animals gaining significantly less weight over time (Figure 1A). In the group submitted to the 8-week protocol, repeated-measures ANOVA has shown again a significant effect of both time [*F*_(8,144)_ = 80.13; *p* < 0.0001] and exposure to CUS [*F*_(1,18)_ = 63.43; *p* < 0.0001] on body weight (Figure 1B). There was also a significant interaction between these factors [*F*_(8,144)_ = 34.17; *p* < 0.0001] with stressed animals gaining significantly less weight over time (Figure 1B). CUS had no significant effect on thymus weight nor on thymic cell number, both in the group exposed to the 4- and the 8-week protocol of CUS (Figures 1C,D). CUS exposure during 4 weeks had no effect on adrenals weight, while exposure to CUS for 8 weeks led to a significant increase on adrenals weight [*t*_(18)=_3.449; *p* = 0.003] (Figures 1E,F). There were no statistically significant changes on corticosterone levels in the group submitted to 4 weeks of CUS, both at *nadir* and *zenith*. Repeated-measures ANOVA has shown a significant effect of exposure to 8 weeks of CUS on corticosterone levels [*F*_(1,18)_ = 21.99; *p* = 0.0002]. *Post hoc* test has shown a statistically significant increase of corticosterone levels in the *zenith* phase of the day, in the group submitted to 8 weeks of CUS [*t*_(18)_ = 4.113; *p* < 0.001] (Figures 1G,H).

### Exposure to 8 weeks of CUS leads to altered emotional behavior but not to memory impairments

**Figure 2:**
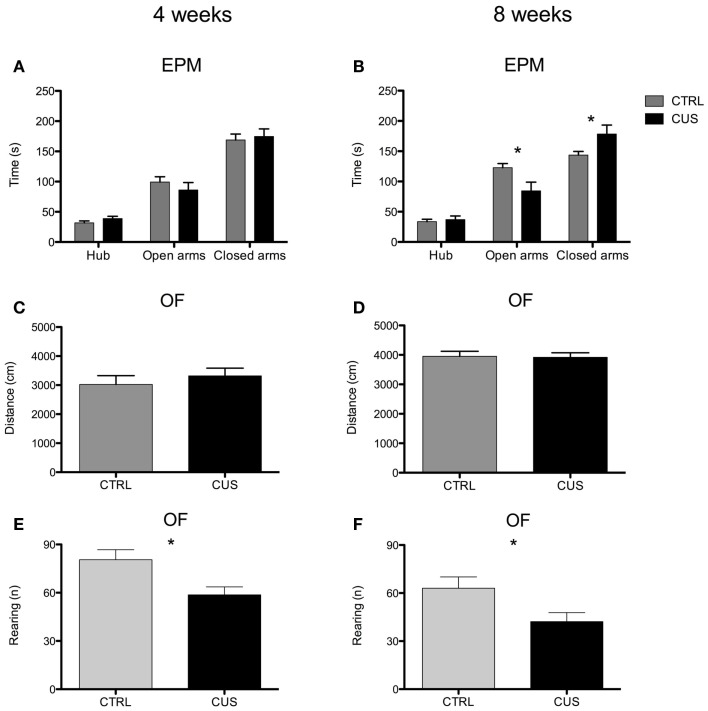
**Impact of 4 and 8 weeks of CUS on anxious-like and locomotor behavior and exploratory activity**. Behavioral performance of mice exposed to 4 **(A)** and to 8 weeks of CUS **(B)** in the EPM. Locomotor function of mice submitted to 4 **(C)** and 8 weeks **(D)** of CUS measured in the OF. Exploratory activity of mice submitted to 4 **(E)** and 8 weeks **(F)** of CUS measured in the OF. Each bar represents the mean ± SEM from 8 to 10 animals per group. *p < 0.05.

There was a significant effect of exposure to 8 but not 4 weeks of CUS on anxious-like behavior, measured by a decreased time spent on the open-arms of the EPM [*t*_(16)_ = 2.401; *p* = 0.029] and an increased time spent in the closed arms [*t*_(16)_ = 2.176; *p* = 0.045] (Figures 2A,B; Figures S1I,J in Supplementary Material) by the 8-week CUS group when compared to controls. Exposure to CUS did not alter locomotor activity, assessed by the OF, both on the group exposed to 4 and 8 weeks of CUS (Figures 2C,D; Figures S1K,L in Supplementary Material), therefore validating behavioral tests that are dependent on an intact locomotor function. CUS had an impact on the exploratory activity, measured by a decrease on the number of rearings in the OF test, both in the group submitted to 4 [*t*_(18)_ = 2.743; *p* = 0.013] (Figure 2E; Figure S1M in Supplementary Material) and 8 weeks of CUS [*t*_(18)_ = 2.308; *p* = 0.033] (Figure 2F; Figure S1N in Supplementary Material).

**Figure 3:**
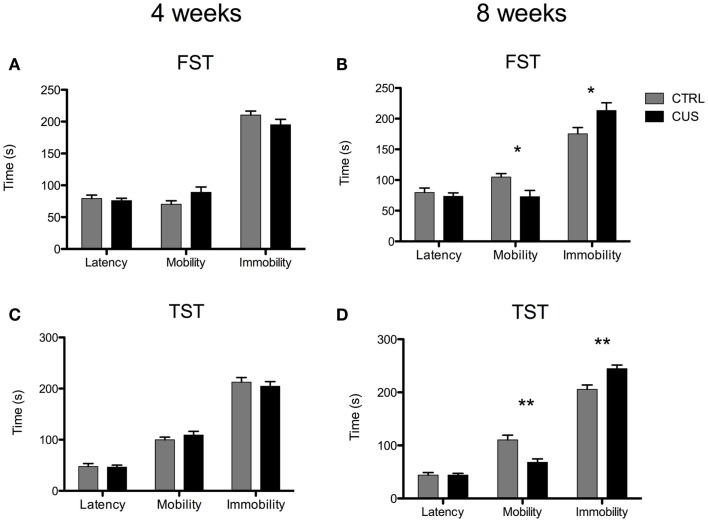
**Impact of 4 weeks vs. 8 weeks of CUS on depressive-like behavior**. Behavioral performance of mice submitted to 4 **(A)** and 8 weeks of CUS **(B)** in the FST. Behavioral performance of mice submitted to 4 **(C)** and 8 weeks of CUS in the TST **(D)**. Each bar represents the mean ± SEM from 9 to 10 animals per group. *p < 0.05, **p < 0.01.

Animals exposed to 4 weeks of stress did not show any major differences in the FST when compared to controls (Figure 3A). The group of animals exposed to 8 weeks of CUS exhibited decreased mobility time [*t*_(18)_ = 2.741; *p* = 0.013] and increased immobility time [*t*_(18)_ = 2.310; *p* = 0.033] in the FST when compared to controls (Figure 3B). In the TST, the group submitted to 4 weeks of CUS did not show any major differences when compared to controls (Figure 3C), while the group submitted to 8 weeks of CUS exhibited a increased immobility time [*t*_(17)_ = 3.710; *p* = 0.002] and an decreased mobility time [*t*_(17)_ = 3.873; *p* = 0.001] (Figure 3D; Figure S1O in Supplementary Material); a typical phenotype of depressive-like behavior. No differences on latency time were found at any time point, both in the FST and TST (Figure 3).

**Figure 4:**
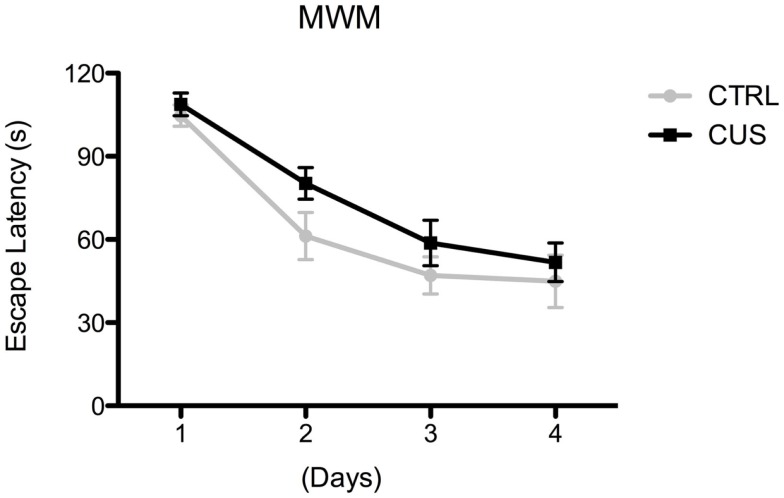
**Impact of 8 weeks of CUS on cognition**. Behavioral performance of mice exposed to 8 weeks of CUS in the MWM. Each point represents the mean ± SEM from 10 animals per group.

The impact of different exposures to CUS was also tested in the MWM task in order to investigate whether the cognitive dimension was also affected. Although there was a slight tendency for a faster learning curve of the control group, especially on day 2 and 3, in comparison to CUS exposed animals, the ANOVA repeated-measures test revealed that there were no significant differences between groups, meaning that, at the end of the learning task, both CUS and control groups were able to successfully learn the task therefore exhibiting an intact spatial learning ability (Figure 4; Figure S1P in Supplementary Material).

### Thymic and splenic cell population changes by exposure to 8 weeks of CUS

**Figure 5:**
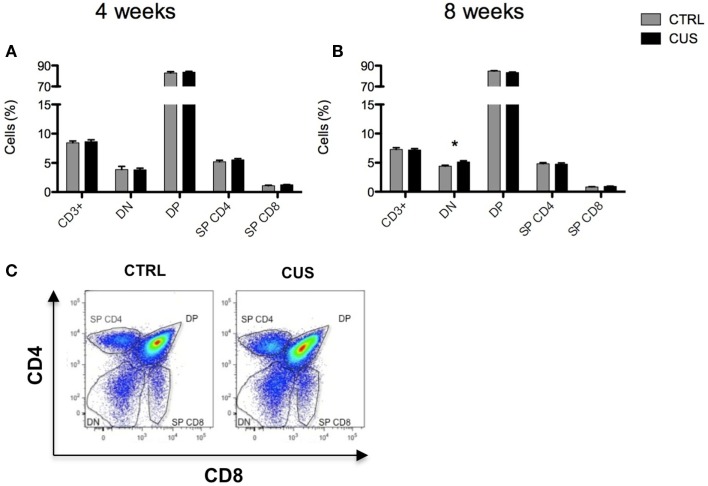
**Impact of 4 weeks vs. 8 weeks of CUS on thymocyte subsets**. Percentage of main cell populations in thymus after exposure to 4 **(A)** and 8 **(B)** weeks of CUS. Flow cytometry plot showing the gating strategy for thymocyte subsets (gate) **(C)**. DN, double-negative thymocytes; DP, double-positive thymocytes; and SPCD4 and SPCD8, single-positive CD4 and CD8 thymocytes, respectively. Each bar represents the mean ± SEM from 10 animals per group. *p < 0.05.

It is known that thymocytes are sensitive to stress hormones, such as glucocorticoids, which modulate several processes along their differentiation within the thymus (32). Due to this well-known susceptibility to stress hormones, the thymus weight, and cellularity have been widely used as indirect measures of stress. Thymocytes might be divided in four main differentiation populations depending on the expression of the CD4 and CD8 co-receptors (CD4^−^CD8^−^ double-negative – DN; CD4^+^CD8^+^ double-positive – DP; CD4^+^CD8^−^ single-positive CD4 – SPCD4; and CD4^−^CD8^+^ single-positive CD8 – SPCD8 cells). We therefore studied the major thymic subsets to determine if our CUS protocols had a differential impact on them. We observed that 4 weeks of CUS did not alter the proportion of the four main thymocyte subsets (Figure 5A) while 8 weeks of stress led to an increase of the DN thymocytes proportion [*t*_(18)_ = 2.681; *p* = 0.020] (Figures 5B,C).

**Figure 6:**
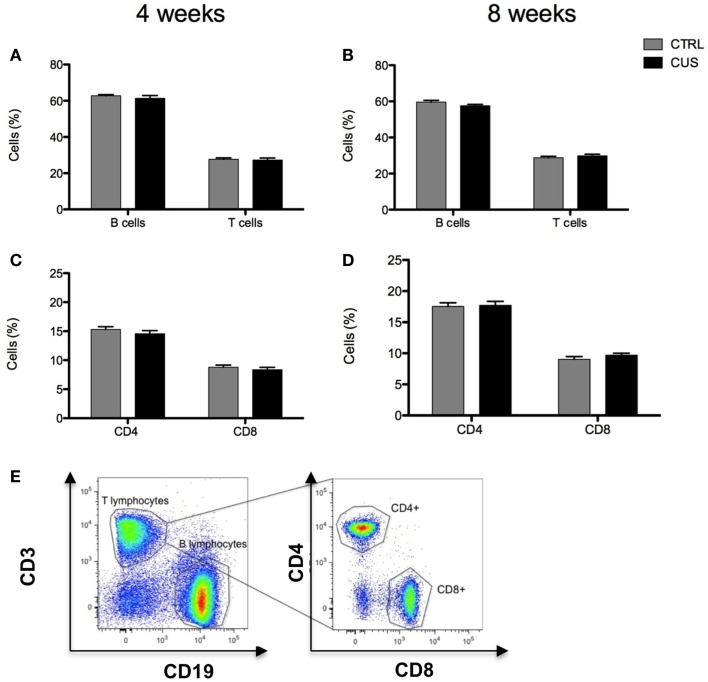
**Impact of 4 vs. 8 weeks of CUS on lymphoid cellular populations in the spleen**. Percentage of T and B cells in spleen after exposure to 4 **(A)** and 8 **(B)** weeks of CUS. Percentage of CD4^+^ and CD8^+^ T cells in spleen after exposure to 4 **(C)** and 8 **(D)** weeks of CUS. Flow cytometry plot showing the gating strategy for T and B-lymphocytes (gates) **(E)**. Each bar represents the mean ± SEM from eight animals per group.

**Figure 7:**
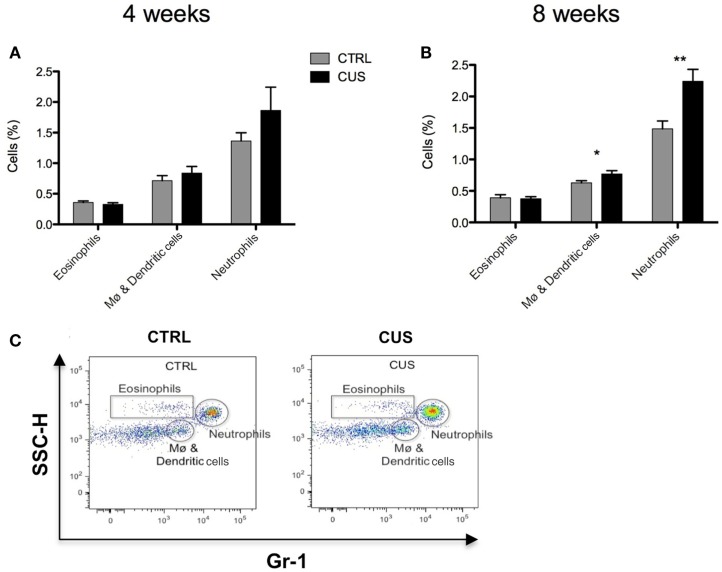
**Impact of 4 vs. 8 weeks of CUS on myeloid cellular populations in the spleen**. Percentage of eosinophils, macrophages/dendritic cells, and neutrophils in spleen after exposure to 4 weeks **(A)** and 8 **(B)** weeks of CUS. Flow cytometry plot showing the gating strategy of myeloid splenocytes subsets (gates) **(C)**. Each bar represents the mean ± SEM from 8 animals per group. *p < 0.05, **p < 0.01.

Since prolonged stress is known to influence the peripheral immune system we consider of relevance to investigate potential alterations caused by CUS on major lymphoid cell populations in the spleen, one of the most important lymphoid organs of the immune system. Animals exposed to 4 or 8 weeks of CUS did not show any differences on the percentage of splenic T and B cells (Figures 6A,B and **E**) nor in the CD4^+^ and CD8^+^ subsets among the T cells (Figures 6C–E). On the contrary, while animals exposed to 4 weeks of CUS did not show any major differences on the percentage of splenic eosinophils, neutrophils, and macrophages/dendritic cells (Figure 7A), the 8-week long CUS protocol led to an increased percentage of macrophages/dendritic cells [*t*_(14)_ = 2.188; *p* = 0.046] and neutrophils [*t*_(14)_ = 3.327; *p* = 0.005] in the spleen (Figures 7B,C).

## Discussion

In the present work, we have optimized a CUS protocol that results in a consistent stress-response in C57BL/6 mice. Published protocols on how to induce chronic stress on rodents are diverse and generate inconsistencies in their behavioral and immunological outcomes (33). Among the main reasons for such inconsistencies are strain inherent differences of stress susceptibility/resistance to distinct protocols. In mice, specifically, the C57BL/6 strain seems to be more resistant to CUS than other strains and/or other species (20–25). Yet, it is by far the most used mouse strain for genetic manipulations. This, and the fact that unpredictable chronic stress exposure is often used as a model of neuropsychiatric disorders, renders an effective CUS protocol in C57BL/6 mice, such as the one herein described, an important addition to the field.

Besides strain considerations, the type, diversity of stressors applied, and stress exposure length are also critical determinants of the impact of chronic stress. Some protocols use a single stressor, e.g., 6 h of daily restraint stress for a 4-week period (34, 35), which, despite being simpler to apply, have several limitations due to lack of unpredictability or the prolonged removal of animals from their homecages with no access to food or water for half of their resting period. On the other hand, reducing restraint stress to 1 h per day in order to overcome this difficulty results in a mild stress protocol.

Other widely used chronic stress protocol consists in the exposure to repeated bouts of social defeat stress, which have shown to induce a stressed phenotype in some C57BL/6 mice. However, both restraint and social defeat stress paradigms are characterized by repeated exposure to a single stressor, which lacks the variability of psychological and physical stressors generally encountered in life. Taking the aforementioned into account, we designed a CUS protocol, based on the appliance of a variety of stressors, presented randomly once per day, in an intermittent and unpredictable fashion, mimicking the variability of stressors encountered on everyday life (construct validity). Although not often used in mice, CUS protocols are widely used in rats and were shown to be highly effective in inducing a stress-related phenotype (6, 11, 12, 36). In addition, by extending this protocol to 8 weeks, instead of the usual 4 weeks, we were able to reach the point where this particular strain of mice clearly and consistently exhibits a maladaptive response to chronic stress with behavioral and immunological alterations (face validity).

One of the main advantages of this 8-week long CUS protocol is that there is no stressor that implies the disturbance of food and/or water consumption, which is of particular importance for metabolism studies, for example. Moreover, in this protocol FST or TST are not used as stressors, as used in some published protocols (9), which means that in our protocol these tests can still be used as behavioral measures.

Reduction on body weight gain, thymic involution (37–39), and increased adrenals weight (40) are typically used as markers of stressors efficacy. We have observed that although behavioral and immunological alterations were only evident after exposing mice to 8 weeks of stress, suppression of body weight gain was observed as early as after 2 weeks of exposure and was maintained throughout the duration of CUS. These findings suggest that, as a read-out of the maladaptive response to stress, body weight gain has a lower threshold than other changes and is not a good marker of the stress-impact in behavior and/or immunity. Moreover, we did not observe a consistent reduction on thymus weight; although we cannot discard the possibility of being unable to detect small differences of thymus weight, specially given that mice were previously transcardially perfused with 0.9% saline. Nevertheless, the concomitant lack of differences in thymic cellularity favors our observation that, in C57BL/6 mice, our CUS protocol does not impact thymus weight significantly. This observation strengths the idea that C57BL/6 are more resistant to the effects of chronic stress than other mouse strains.

An overactive hypothalamic–pituitary–adrenal (HPA) axis is also a feature of a maladaptative response to chronic stress (41). In fact, resistance to chronic stress can be associated with an effective negative feedback system that is able to shut down the excessive production of glucocorticoids occurring in response to stress (42). We observed that the 8-week long CUS protocol was the only one that led to a persistent increase on circulating corticosterone levels and increased adrenals weight, features consistent with a hyperactive HPA axis. Of note, based on corticosterone levels at zenith we identified a reduced number of resistant animals (2 out of 10 in one of the experiments and 2 out of 10 in the replicated experiment), a proportion of stress-resistance very similar to what already have been described in other models of chronic stress (26). Accumulating evidence shows that glucocorticoids modulate the behavioral effects of chronic stress (43, 44). In accordance, we observed that the 8-week long CUS protocol, the only that induced a hyperactive HPA axis, had a negative impact on emotional behavior. Specifically, we observed an enhanced anxious-like behavior, revealed by an increased time spent in the closed arms, and a decreased time in the open arms of the EPM. These animals also displayed behavioral despair, a symptom of depressive-like behavior, as they spent more time immobile in the FST. Of notice, this was further confirmed by performing the TST, another validated test for depressive-like behavior assessment.

Despite the emotional changes caused by 8 weeks of CUS exposure, cognitive functioning, namely spatial learning, seems to be intact, confirming data from other model of chronic stress (uCMS) (10). In fact, we observed that although stressed animals at the end of 4 days of MWM training were able to learn task at the same level as controls, there was a tendency for a slower learning progression on day 2 and 3. This type of learning pattern was previously shown using rats submitted to CUS (11), therefore emphasizing that the effects of CUS on spatial learning are more subtle than those on emotional behaviors. Contrary to the above mentioned effects, chronic stress triggers a decreased exploratory behavior of mice, both at 4 and 8 weeks of CUS, which might not be dependent on increased levels of corticosterone.

Although we cannot completely discard the possible confounder effect from performing two behavioral tests in the same day, we believe that data from the OF and TST was not significantly affected by acute stress caused by prior testing; indeed motor function (measured by OF) is not known as a target of acute stress, whereas data from TST were confirmed by the findings of the FST.

Glucocorticoids play a crucial role on thymopoiesis (32, 39), a process that occurs in the thymus in which immature precursor cells differentiate into mature T cells. In accordance, it was previously shown that rats exposed to chronic stress, with increased levels of circulating corticosterone levels, exhibit an increase in the percentage of DN thymocytes, while the percentage of SPCD4 was decreased (39). In our model, an increase in the percentage of DN thymocytes was observed. Still, contrary to the previously described (39), we did not observe any differences on the SPCD4 and SPCD8 populations of thymocytes, which may be due to the stress-resistance inherent to this particular strain of mice. T and B-lymphocytes in the spleen were not altered by exposure to chronic stress. However, we observed that exposure to 8 weeks of CUS (and not to 4 weeks) led to alterations in the cell composition of the spleen, characterized by an increased percentage of myeloid cells (macrophages/dendritic cells and neutrophils), in agreement with previous reports in both mice (45, 46) and humans (46). Glucocorticoids were shown to inhibit neutrophils’ apoptosis, which may explain the persistent presence of these cells with short life span (47) in the spleen of chronically stressed animals. Moreover, it was shown that stress, through norepinephrine signaling from sympathetic nerve fibers, increased the proliferation of hematopoietic progenitors in the bone marrow giving rise to an increase on disease-promoting monocytes and neutrophils output (46). Stress was also shown to increase monocyte recruitment to the brain by increased expression of cytokines and chemokines in specific brain regions. And more importantly, this monocyte recruitment to the brain was shown to be essential for the development of anxiety behavior induced by stress (45).

The absence of neuroendocrine, major behavioral and immunological alterations seen in the 4-week CUS exposed group could reflect the temporal dynamics of the stress-response rather than a failure to respond to stress. In fact, it should be noted that stress did impact the body weight gain and exploratory behavior on this group. This absence of major alterations resembles the Hans Selye’s resistance phase of the so-called “syndrome of adaptation” (48) in which adaptative processes reinstall homeostasis during stress, including the normalization of glucocorticoid secretion. Therefore, the 4-week CUS protocol may be preferable to studies that target this specific stage of the stress-response like for example those that want to show a negative impact of a particular treatment on the stress-response, since the 8-week CUS alterations may approach a “ceiling effect.” In contrast, the alterations observed in the 8-week version of CUS are consistent with phase 3 of this syndrome, where the system is no longer able to cope with stressors and is exhausted, which renders this version a robust model of the maladaptative response to chronic stress.

The establishment of a robust mouse model of stress-related disorders on C57BL/6 background represents a valuable research tool endowing the study of different genetic contributions to chronic stress-responses, which may enhance current knowledge on the neurobiology and immunology of complex neuropsychiatric and other stress-related disorders.

## Conflict of Interest Statement

The authors declare that the research was conducted in the absence of any commercial or financial relationships that could be construed as a potential conflict of interest.

## Supplementary Material

The Supplementary Material for this article can be found online at http://www.frontiersin.org/Journal/10.3389/fpsyt.2015.00006/abstract
