# Supplementary material for: An Efficient Chronic Unpredictable Stress Protocol to Induce Stress-Related Responses in C57BL/6 Mice
Source: Front Psychiatry. 2015 Feb 2;6:6. doi: 10.3389/fpsyt.2015.00006 (PMC4313595; doi:10.3389/fpsyt.2015.00006)
Supplement: Supplementary file 1 [file Data_Sheet_1.DOCX]

***Supplementary Material***

**An efficient chronic unpredictable stress protocol to induce stress-related responses in C57BL/6 mice**

**Monteiro, S.^1,2^, Roque, S.^1,2^, de Sá-Calçada, D.^1,2^, Sousa, N.^1,2^, Correia-Neves, M.^1,2^, Cerqueira, J.J.^1,2^***

^1^Life and Health Sciences Research Institute (ICVS), School of Health Sciences, University of Minho, Braga, Portugal

^2^ICVS/3B’s – PT Government Associate Laboratory, Braga/Guimarães, Portugal

*** Correspondence:** João José Cerqueira, Life and Health Sciences Research Institute (ICVS) , School of Health Sciences, University of Minho, Campus de Gualtar, ICVS/3B's - PT Government Associate Laboratory, Campus de Gualtar 4710-057 Braga, Portugal , jcerqueira@ecsaude.uminho.pt

**Supplementary Data**

The full data set of the replicated experiment is depicted below (supplementary Figure 1). We also added a table with the stressors sequence and layout of the replication experiment (supplementary Table 1).

1. **Supplementary Figures and Tables**

## Supplementary Figures





**Supplementary Figure 1. Replication experiment data set.** Body weight gain for animals submitted to 4 (A) and 8 (B) weeks of CUS. Thymus weight after exposure to 4 (C) and 8 (D) weeks of CUS. Adrenals weight after 4 (E) and 8 weeks of CUS (F). Corticosterone levels in the serum of animals from the group submitted to 4 (G) and 8 (H) weeks of CUS. Behavioral performance of mice exposed to 4 (I) and to 8 weeks of CUS (J) in the EPM. Locomotor function of mice submitted to 4 (K) and 8 weeks (L) of CUS measured in the OF. Exploratory activity of mice submitted to 4 (M) and 8 weeks (N) of CUS measured in the OF. Behavioral performance of mice submitted to 8 weeks of CUS in the TST (O) and MWM (P). *p<0.05, ** p<0.01, *** p<0.001

Body weight gain, *post-mortem* thymus and adrenal weight, serum levels of corticosterone, performance in the elevated plus maze (EPM), open field (OF), tail suspension test (TST) and morris water maze (MWM) were monitored in the replicated experiment to control for stressors efficacy (Supplementary Fig.1). In the group submitted to the 4-weeks protocol of CUS, both time (F_(4,112)_=73.92; p<0.0001) and exposure to CUS (F_(1,28)_=32.59; p=0.0001) had a significant impact on body weight (supplementary Fig. 1A). Moreover, there was a significant interaction between these factors (F_(4,112)_=27.33; p<0.0001) with stressed animals gaining significantly less weight over time (supplementary Fig. 1A). In the group submitted to the 8-weeks protocol, repeated-measures ANOVA has shown again a significant effect of both time (F_(7,182)_=48.68; p<0.0001) and exposure to CUS (F_(1,26)_=19.21; p=0.0001) on body weight (supplementary Fig. 1B). There was also a significant interaction between these factors (F_(7,182)_=9.81; p<0.0001) with stressed animals gaining significantly less weight over time (supplementary Fig. 1B). CUS has slightly decreased the thymus weight, both in the group exposed to the 4- and the 8-weeks protocol of CUS (t_(23)_=2.762, p=0.01; t_(24)_=2.259, p=0.03) (supplementary Fig. 1C and D). CUS exposure had no effect on adrenals weight, while exposure to CUS for 8 weeks led to a tendency for an increase on adrenals weight (supplementary Fig.1E and F). There were no statistically significant changes on corticosterone levels in the group submitted to 4 weeks of CUS, both at *nadir* and *zenith.* Repeated-measures ANOVA has shown a significant effect of exposure to 8 weeks of CUS on corticosterone levels (F_(1,18)_=14.54; p=0.001). Post-hoc test has shown a statistically significant increase of corticosterone levels in the *zenith* phase of the day, in the group submitted to 8 weeks of CUS (t_(18)_=4.956; p<0.001) (supplementary Fig.1G and H).There was a significant effect of exposure to 8 but not 4 weeks of CUS on anxious-like behavior, measured by an increased time spent in the closed arms (t_(22)_=2.418; p=0.024 ) (supplementary Fig. 1I and J) by the 8-weeks CUS group when compared to controls. Exposure to CUS did not alter locomotor activity, assessed by the OF, both on the group exposed to 4 and 8 weeks of CUS (supplementary Fig. 1K and L). CUS had an impact on the exploratory activity, measured by a decrease on the number of rearings in the OF test, both in the group submitted to 4 (t_(27)_=2.122; p=0.043) (supplementary Fig. 1M) and 8 weeks of CUS (t_(25)_=2.086; p=0.047) (supplementary Fig. 1N). In the TST the 8 week CUS exposure has led to an increased time immobile (t_(26)_=3.285; p=0,003) and decreased time mobile (t_(26)_=3.255; p=0.003) (supplementary Fig. 1O). There was no statistically differences observed in the MWM test between exposure to 8 weeks of CUS and controls (supplementary Fig. 1P).

## Suplementary Table

|  | **Mon** | **Tue** | **Wed** | **Thu** | **Fri** | **Sat** | **Sun** |
| --- | --- | --- | --- | --- | --- | --- | --- |
| Week 1 | Bw basal restraint | restraint | shaking | social defeat | hot drier | restraint | tilted cage |
| Week 2 | Bw 1st restraint | shaking | social defeat | hot drier | restraint | social defeat | overnight illumination |
| Week 3 | Bw 2nd hot drier | social defeat | restraint | Blood collect. *zenith* | social defeat | inverted light | shaking |
| Week 4 | Blood collect. nadir Bw 3rd social defeat | EPM&OF restraint | FST&TST Social defeat | shaking | restraint | restraint | hot drier |
| Week 5 | BW 4th week restraint | Sacrifice 4w Tilted cage | Cytometry hot drier | restraint | social defeat | hot drier | inverted light |
| Week 6 | BW 5th week hot drier | social defeat | restraint | inverted light | restraint | social defeat | hot drier |
| Week 7 | BW 6th week restraint | social defeat | restraint | blood collect. zenith | shaking | restraint | overnight illumination |
| Week 8 | BW 7th wk Blood collect.nadir | EPM&OF restraint | FST&TST Social defeat | MWM Shaking | MWM restraint | MWM restraint | MWM Shaking |
| Week 9 | BW 8th wk | Sacrifice 8w | Cytometry |  |  |  |  |

**Supplementary Table 1 – Stressors distribution and layout of the replication experiment.**
